# Plant-Based Diets and Cancer Prognosis: a Review of Recent Research

**DOI:** 10.1007/s13668-022-00440-1

**Published:** 2022-09-23

**Authors:** Luisa Hardt, Yahya Mahamat-Saleh, Dagfinn Aune, Sabrina Schlesinger

**Affiliations:** 1 Institute for Biometrics and Epidemiology, German Diabetes Center, Leibniz Center for Diabetes Research at Heinrich Heine University Düsseldorf, Auf’m Hennekamp 65, 40225 Düsseldorf, Germany; 2 International Agency for Research On Cancer, Lyon, France; 3 Department of Epidemiology and Biostatistics, School of Public Health, Imperial College London, St. Mary’s Campus, Norfolk Place, Paddington, London, UK; 4 Department of Nutrition, Oslo New University College, Oslo, Norway; 5 Department of Endocrinology, Morbid Obesity and Preventive Medicine, Oslo University Hospital, Oslo, Norway

**Keywords:** Cancer survival, Cancer prognosis, Plant-based diets, Whole grain, Vegetable, Nuts

## Abstract

***Purpose of Review*:**

Although plant-based diets are recommended for cancer prevention, their role in cancer survival is still uncertain. The purpose of this systematic review is to summarize the association between postdiagnosis plant-based diets and prognosis in cancer survivors.

***Recent Findings*:**

There is indication that higher intake of plant-based foods was associated with improved prognosis in cancer survivors. For colorectal cancer survival, a better prognosis was observed for a high intake of whole grains and fibre. For breast cancer survival, a higher intake of fruit, vegetable and fibre and a moderate intake of soy/isoflavone were associated with beneficial outcomes. A higher vegetable fat intake was related to improved prognosis in prostate cancer survivors.

***Summary*:**

Emerging evidence suggests benefits of postdiagnosis plant-based diets on prognosis in cancer survivors. However, given the high heterogeneity between studies, further research in cancer survivors, considering clinical factors (e.g. treatment, stage) and methodological aspects (e.g. timing of dietary assessment), is needed.

## Introduction

Due to rising cancer incidence worldwide combined with improved survival rates especially in high-income countries as a result of advances in treatment and early detection in the past decades, the number of people living with or after cancer is growing [1]. According to current estimates, there were 50.5 million people living with cancer in 2020 who had been diagnosed within the last 5 years, and this number is projected to further increase in the next years [2]. A cancer diagnosis is recognized as a “teachable moment” [3] and is frequently observed to trigger lifestyle changes, including changes in dietary habits, in hope of improving cancer prognosis and overall health [4–6]. To date, however, there are no specific recommendations, and cancer prevention recommendations for the general population are applied to cancer survivors, although they may represent a specific target group with different nutritional needs and metabolic functions [7, 8]. Plant-based diets are an integral part of evidence-based recommendations for primary prevention of cancer and other non-communicable diseases (NCDs), promoting a diet rich in whole grains, vegetables, fruit, nuts and legumes and a limited consumption of red and processed meat [9]. As cancer survivors are at elevated risk of premature death mainly due to their primary cancer, but also due to second primary cancers and other comorbidities, such as coronary heart disease, obesity and diabetes mellitus, these guidelines might be even more important to them than for people without a history of cancer [10]. Recent studies have investigated associations between dietary factors and survival or prognosis of various cancers, and findings indicated beneficial outcomes for higher intake of plant-based diets and components in cancer survivors [11••, 12•, 13•, 14••, 15•, 16]. In this context, it is important to consider several aspects, including the timing of dietary assessment (e.g. assessment after cancer diagnosis = postdiagnostic diet), different cancer sites (e.g. breast, colorectal, prostate cancer) and specific prognostic outcomes, such as cancer-specific mortality, recurrence or overall survival. To provide an overview on this topic, we conducted a systematic review of recently published meta-analyses and prospective studies investigating the association between postdiagnostic plant-based diets and overall and site-specific cancer prognosis.

## Search Strategy and Study Selection

We conducted a systematic search of the recently published literature on the association between postdiagnosis plant-based diets and foods with cancer prognosis in individuals after a cancer diagnosis in PubMed from January 1, 2015 to November 9, 2021. We did not apply any restrictions or filters and used predefined search terms. We used MeSH terms and title/abstract text words related to plant-based dietary patterns (i.e. vegan, vegetarian, plant-based), food groups and foods (i.e. fruit, vegetables, whole grains, nuts, legumes, seeds, plant oils, vegetable products, soya, olive oil, tofu), nutrients or other bioactive compounds (i.e. plant protein, folate, carotenoids, polyphenols, isoflavones, glucosinolates, fibre) and cancer (i.e. cancer tumour, carcinoma), combined with prognostic outcomes (i.e. prognosis, overall survival, all-cause mortality, cancer-specific mortality/survival, recurrence), related to the study population (i.e. cancer survivors) and the study design (i.e. prospective cohort studies, systematic reviews, meta-analyses). We excluded cross-sectional and retrospective case–control studies as well as studies on prediagnosis diet. Screening of studies was conducted by at least two independent researchers. The reference lists of selected articles were additionally hand-searched for relevant literature.

## Search Results

**Fig. 1:**
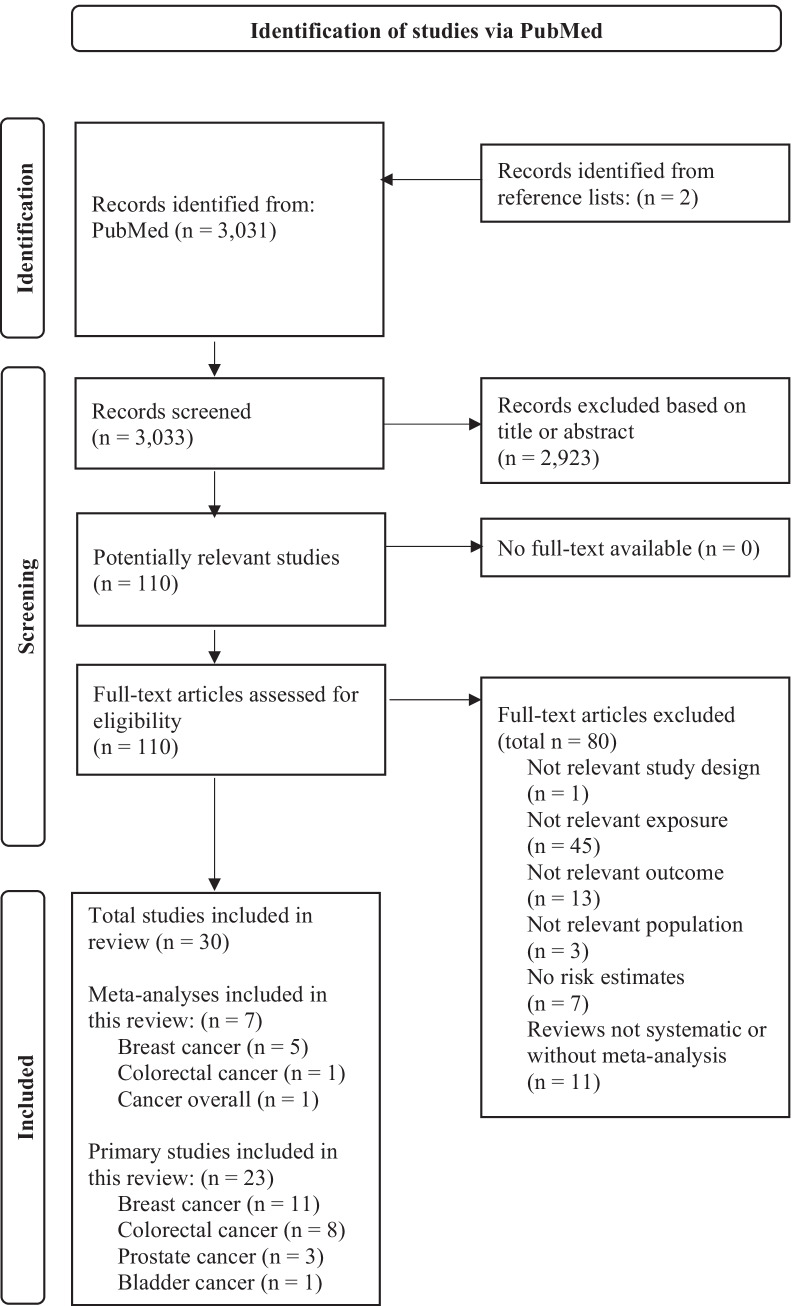
Adapted flow-chart of the study selection process according to PRISMA 2020 statement [44]

In total, 30 studies (seven meta-analyses and 23 primary studies) met our inclusion criteria (Fig. 1). Five of the 23 recently published primary studies were already considered in the identified meta-analyses, and the remaining 18 primary studies were additionally identified through the systematic literature search (Table 1). Of the seven meta-analyses [7, 17, 18, 19••, 20••, 21, 22••], five focused on breast cancer (BC) [17, 18, 20••, 21, 22••], one on colorectal cancer (CRC) [19••] and one on overall cancer [7]. The meta-analysis on overall cancer included prospective cohort studies (Table 1).

**Table 1:** Overview of the identified meta-analyses (*n* = 7) published between 2015 and November 2021 that investigated the associations between plant-based diets and components with cancer prognosis according to cancer sites

Reference	Exposure	*n*	Outcome	SHR (95% CI) for each outcome High vs. low analysis	I^2^ (%)	Prognosis	Studies included in meta-analysis
**Cancer overall (*n* = 1 meta-analysis)**							
**Schwedhelm et al. **[7]	Fruit	3	AC-M	0.96 (0.64–1.45)	49	↔	Beasley et al. [45] (BC) Sandoval et al. [46] (oral cancer), Shen et al. [47] (nasopharyngeal cancer)
	Vegetable	4	AC-M	0.88 (0.59–1.30)	78	↔	Beasley et al. [45] (BC), Holmes et al. [48] (BC), Nechuta et al. [49] (BC), Sandoval et al. [46] (oral cancer)
**Breast cancer (*n* = 5 meta-analyses)**							
**He et al. **[17]	Vegetable	2	OS	0.95 (0.73–1.24)	17	↔	Pierce et al. [50] McCullough et al. [28]
	Fruits	3	OS	1.04 (0.77–1.42)	41	↔	Holmes et al. [48] Pierce et al. [50] Beasley et al. [45]
	Vegetables	3	OS	1.08 (0.75–1.55)	60	↔	Holmes et al. [48] Pierce et al. [50] Beasley et al. [45]
	Cruciferous vegetables	2	OS	1.03 (0.90–1.17)	0	↔	Beasley et al. [45] Nechuta et al. [49]
**Peng et al. **[21]	Total vegetable	2	AC-M	1.05 (0.60–1.85)	76	↔	Beasley et al. [45] Holmes et al. [48]
	Fruit	2	AC-M	0.82 (0.29–2.36)	87	↔	No information
**He et al. **[18]	β-carotene	3	OS	1.06 (0.88–1.29)	0	↔	Holmes et al. [48] Beasley et al. [45] Greenlee et al. [51]
		2	BCSS	1.17 (0.91–1.51)	0	↔	Beasley et al. [45] Greenlee et al. [51]
	Lycopene	3	OS	0.96 (0.73–1.27)	30	↔	Holmes et al. [48] Beasley et al. [45] Greenlee et al. [51]
		2	BCSS	1.12 (0.85–1.48)	0	↔	Beasley et al. [45] Greenlee et al. [51]
	α-carotene	2	OS	1.01 (0.82–1.25)	0	↔	Holmes et al. [48] Beasley et al. [45]
	β-cryptoxanthin	2	OS	1.04 (0.72–1.50)	65	↔	Holmes et al. [48] Beasley et al. [45]
**Jayedi et al. **[20••]	Fibre	3	AC-M	0.70 (0.55–0.89)	0	↓	Beasley et al. [45] Belle et al. [52] Holmes et al. [48]
		3	BC-M	0.72 (0.51–1.01)	0	(↓)	Beasley et al. [45] Belle et al. [52] Holmes et al. [48]
**Qiu et al. **[22••]	Soy and isoflavones	2	OS	0.80 (0.62–1.04)	24	(↓)	Nechuta et al. [40] *Zhang et al. *[31]
**Colorectal cancer (*n* = 1 meta-analysis)**							
**Hoang et al. **[19••]	Whole grain	3	ACM	0.83 (0.69–0.99)	0	↓	*Brown et al. *[12•] *Song et al. *[15•] *Van Blarigan et al. *[35]

↓ decreased relative risk, ↑ increased relative risk, ↔ no association, () tendency: the null effect is included in the 95% CI; italicized studies are also included as primary studies in the present review

*n* number of studies, *SHR* summary hazards ratio, *95% CI* 95% confidence interval, *OS* overall survival, *AC-M* all-cause mortality, *BCSS* breast cancer specific survival, *BC-M* breast cancer-specific mortality, *Rec* recurrence, *CRC-M* colorectal cancer-specific mortality

**Table 2:** Prospective cohort studies (*n* = 23) published between 2015 and November 2021 that investigated the associations between plant-based diets and their selected key components and cancer prognosis according to cancer sites

Reference, region, cohort	Population		Time since cancer diagnosis			FU			Dietary assessment			N of cases			Adjustments		Exposure(s)			HR (95% CI) for each outcome (highest vs lowest or dose response)							
**Breast cancer (*n* = 11)**																											
**Andersen et al. **[23] Denmark, The Danish Diet, Cancer and Health (DCH) cohort	977 post-menopausal women, mean age at diagnosis: 66 y		n.a			7 y			Semi-quantitative FFQ			175 deaths, 121 due to BC, 152 recs			Pre-diagnostic intake of the variable of interest, Age and year at diagnosis, time since diagnosis, alcohol intake, smoking, physical activity, BMI, educational level, tumor size, nodal status, ER status, post-diagnostic diet		Intake of WG per serving/d			Rec			BC-M				AC-M
																	Rye bread/50 g			1.16 (0.84–1.56)			1.25 (0.96–1.62)				1.15 (0.92–1.43)
																	WG bread/40 g			0.82 (0.65–1.05)			1.02 (0.84–1.24)				0.95 (0.80–1.12)
																	Oatmeal/muesli/50 g			0.94 (0.68–1.42)			0.88 (0.60–1.30)				0.88 (0.63–1.22)
																	Total WG (∑)/50 g			0.94 (0.79–1.13)			1.06 (0.91–1.23)				1.00 (0.88–1.14)
**Anyene et al. **[11••] USA, the Pathways Study	3646 women with BC (stages 1–4), 71% post-menopausal mean age at diagnosis: 60 y		2.3 m			9.5 y for death, 9.2 y for rec			Semi-quantitative FFQ (at 6-, 24-, and 72-m FU)			461 recs, 653 deaths, 323 due to BC, 330 non-BC deaths			Age at diagnosis, total energy intake, physical activity, race/ethnicity, education, menopausal status, smoking status		3 plant-based indices			Rec		AC-M			BC-M		Non-BC-M
																	PDI (per 10-point increase)			1.17 (0.98–1.39)		0.96 (0.82–1.11)			0.98 (0.79–1.22)		0.90 (0.73–1.11)
																	hPDI (per 10-point increase)			1.11 (0.97–1.26)		0.93 (0.83–1.05)			1.07 (0.91–1.25)		0.83 (0.71–0.98)
																	uPDI (per 10-point increase)			0.90 (0.79–1.03)		1.07 (0.96–1.20)			0.94 (0.80–1.10)		1.20 (1.02–1.41)
**Farvid et al. **[24] USA, Nurses’ Health Study (NHS) and NHS II	8932 women with invasive BC, stages I–III, 99% post-menopausal		At least 12 m			11.5 y			Validated, semi-quantitative FFQ, every 4 y after diagnosis			2532 deaths, 1017 due to BC			Age and calendar year at diagnosis, time between diagnosis and first FFQ, pre-diagnostic BMI, BMI change after diagnosis, smoking, physical activity, oral contraceptive use, alcohol consumption, total energy intake, pre-diagnostic menopausal status, age at menopause, post-menopausal hormone use, aspirin use, race, stage of disease, ER-, PR-status, radiotherapy, chemotherapy		Carbohydrate intake from… (Q5 vs. Q1)			BC-M					AC-M		
																	Fruit			1.02 (0.83–1.25)					0.97 (0.85–1.11)		
																	Fruit juice			1.24 (1.02–1.50)					1.15 (1.01–1.30)		
																	Vegetables			0.84 (0.69–1.02)					0.86 (0.75–0.97)		
																	Whole grains			1.12 (0.91–1.37)					0.92 (0.80–1.05)		
																	Refined grains			0.96 (0.79–1.18)					1.16 (1.02–1.32)		
																	Legumes			1.12 (0.92–1.36)					0.99 (0.88–1.13)		
																	Potatoes			1.25 (1.02–1.52)					1.13 (0.99–1.28)		
**Farvid et al. **[13•] USA, Nurses’ Health Study (NHS) and NHS II	8927 women with invasive BC, stages I–III, 99% post-menopausal		At least 12 m			11.5 y			Validated, semi-quantitative FFQ, every 4 y after diagnosis			2521 deaths, 1070 due to BC			Age and calendar year at diagnosis, time between diagnosis and first FFQ, pre-diagnostic BMI, BMI change after diagnosis, smoking, physical activity, oral contraceptive use, alcohol consumption, total energy intake, pre-diagnostic menopausal status, age at menopause, post-menopausal hormone use, aspirin use, race, stage of disease, ER-, PR- status, radiotherapy, chemotherapy,		Fruit and vegetable intake			BC-M					AC-M		
																	Total fruits + vegetables Q5 vs. Q1 Per 2 servings/d			0.88 (0.71–1.09) 0.98 (0.90–1.06)					0.82 (0.71–0.94) 0.93 (0.88–0.98)		
																	Total fruits Q5 vs. Q1 Per 2 servings/d			1.03 (0.83–1.26) 1.01 (0.85–1.19)					0.93 (0.81–1.07) 0.93 (0.83–1.03)		
																	Total vegetables Q5 vs. Q1 Per 2 servings/d			0.87 (0.70–1.08) 0.94 (0.84–1.05)					0.84 (0.72–0.97) 0.89 (0.82–0.95)		
																	Fruit juices Q5 vs. Q1			1.33 (1.09–1.63)					1.19 (1.04–1.36)		
																	Cruciferous vegetables Q5 vs. Q1			1.02 (0.83–1.24)					0.87 (0.76–0.99)		
																	Vegetables high in β-carotene Q5 vs. Q1			0.90 (0.73–1.10)					0.80 (0.70–0.91)		
																	Vegetables high in α-carotene Q5 vs. Q1			1.14 (0.93–1.40)					1.01 (0.89–1.16)		
**Ho et al.[ **[25•] China, The Hong Kong Breast Cancer Survival Study (HKBCSS)	1460 women with invasive BC, stages I–III, mean age at diagnosis: 52 y, 48% post-menopausal		3 m and 21 m			71 m			Validated semi-quantitative FFQ with a validated 29-item soy FFQ at baseline and at 18-month FU			71 deaths, 64 due to BC, 137 recs			Age, educational level, menopausal status, cancer stage, comorbidity, ER-status, PR-status, hormonal therapy, radio therapy * + HER2 status ** age, educational level, menopausal status, cancer stage, ER status, PR status, hormonal therapy		Soy isoflavone intake^a^ (mg/1000 kcal)			AC-M			BC-M*				Rec**
																	Q2 vs. Q1 Q3 vs. Q1 Q4 vs. Q1			0.49 (0.25–0.97) 0.44 (0.22–0.89) 1.15 (0.63–2.10)			0.45 (0.21–0.93) 0.49 (0.23–1.01) 1.24 (0.66–2.32)				0.60 (0.36–0.99) 0.78 (0.48–1.26) 1.21 (0.76–1.93)
**Holmes et al. **[26] USA, Nurses’ Health Study	6348 women with invasive BC, stages I–III, 99% post-menopausal		At least 12 m			n.a			Validated semi-quantitative FFQ, every 4 y			1046 distant recs, 1847 deaths, 919 due to BC			Age and calendar year at diagnosis, time since diagnosis, energy intake, BMI, weight change, age at first birth, parity, oral contraceptive use, menopausal status, hormone therapy use, aspirin use, alcohol, smoking, physical activity, tumor stage, treatment, animal protein intake		Vegetable protein intake			Distant Rec			BC-M				AC-M
																	Q5 vs. Q1			1.20 (0.97–1.49)			1.09 (0.87–1.37)				0.97 (0.83–1.14)
**Jaskulski et al. **[27•] Germany, Mamma Carcinoma Risk factor Investigation (MARIE) study	1686 post-menopausal women with invasive BC (non-metastatic) or in situ carcinoma, median age at diagnosis: 63 y, mean age at dietary assessment: 69 y		6.4 y			5.5 y			Blood draw, phyto-estrogen metabolite conc. measured UPLC-MS/MS according to a validated method			142 deaths, 73 due to BC, 93 recs			Age at diagnosis, time between blood draw at baseline and at FU1, study centre, tumor size, affected lymph nodes, grade, ER- and PR-status, mode of detection, recs between diagnosis and blood draw		8 circulating phytoestrogen metabolites (Cont. ^b^)			AC-M			BC-M				Rec
																	Enterolactone			0.98 (0.86–1.11)			1.05 (0.87–1.26)				1.14 (0.98–1.33)
																	Genistein			0.94 (0.83–1.08)			0.93 (0.77–1.13)				1.17 (1.01–1.36)
																	Daidzein			1.00 (0.90–1.12)			1.00 (0.85–1.18)				1.02 (0.88–1.17)
																	Formonetin			1.09 (0.90–1.32)			1.17 (0.90–1.51)				0.86 (0.64–1.15)
																	Naringenin			0.99 (0.87–1.13)			0.99 (0.81–1.22)				1.08 (0.94–1.25)
																	Resveratrol			1.10 (0.96–1.27)			1.09 (0.89–1.33)				1.19 (1.02–1.40)
																	Kaempferol			0.91 (0.68–1.21)			0.78 (0.51–1.19)				0.77 (0.55–1.09)
																	Luteolin			1.18 (0.77–1.80)			1.96 (1.07–3.58)				1.20 (0.68–2.12)
**Mc Cullough et al. **[28] USA, Cancer Prevention Study II (CPS II) Nutrition Cohort	2152 women with BC (stages I–III), mean age at diagnosis: 71 y		3.3 y			9.9 y			152-item Harvard FFQ, post-diagnostic diet was evaluated based on the first FFQ at least 1 y after diagnosis			640 deaths, 192 due to BC, 129 due to CVD, 319 due to other causes			Age at diagnosis, diagnosis year, tumor stage and grade, ER and PR status, treatment, BMI, smoking status, physical activity, energy intake, other dietary factors		Adherence to dietary ACS recommends for cancer prevention			BC-M		CVD-M			OC-M		AC-M
																	Fruit and vegetable intake score (Q4 vs. Q1)			1.31 (0.83–2.06)		0.80 (0.45–1.44)			0.93 (0.65–1.34)		1.03 (0.80–1.33)
																	% WG from total grains (Q4 vs. Q1)			1.24 (0.81–1.88)		1.43 (0.82–2.50)			0.91 (0.64–1.29)		1.09 (0.86–1.38)
**McEligot et al. **[29] USA, Cancer Surveillance Program of Orange Country (CSPOC)	471 post-menopausal women with primary BC (stages 0–IV) 15% in situ, 1% metastatic, mean age at diagnosis: 64 y		1.2 y			6.7 y			Blood draw, folate conc. measured by isotope-dilution LC–MS/MS			85 deaths			Cancer stage, age at diagnosis, BMI, parity, HRT use, treatment, alcohol intake, folic acid supplement use, energy intake		Plasma total folate conc			OS							
																	Q4 vs. Q1			0.41 (0.19–0.90)							
**Wang et al. **[30•] China, Shanghai Breast Cancer Survival Study (SBCSS)	3449 long-term BC survivors, stages I–IV, mean age at diagnosis: 55 y		5 y			8.3 y			Validated, semi-quantitative FFQ			374 deaths, 209 DFS events			Age at diagnosis, total energy intake, income, education, TNM stage, ER and PR status, menopause age, physical activity, Chinese Food Pagoda 2007 score, soy food intake, BMI and weight change during first 5-year FU		Nut intake			OS					DFS		
																	< Median ≥ Median (vs. non-consumer)			1.00 (0.73–1.38) 0.74 (0.52–1.05)					0.55 (0.37–0.81) 0.48 (0.31–0.73)		
																	Peanuts Walnuts Other nuts			0.85 (0.63–1.20) 0.82 (0.59–1.13) 0.82 (0.59–1.14)					0.50 (0.34–0.74) 0.46 (0.31–0.69) 0.51 (0.34–0.76)		
**Zhang et al. **[31] North America, The Breast Cancer Family Registry (BCFR)	1466 women with invasive BC, 51% postmenopausal at enrolment, mean age 52 y		Within 5 y post-diagnosis			9.4 y			Validated, semi-quantitative FFQ			261 deaths			Age, study site, total caloric intake, race/ethnicity, education, total fiber intake, Healthy Eating Index, treatment, recreational physical activity, BMI, alcohol intake, smoking status		Total isoflavone intake (mg/d)			AC-M							
																	Q4 vs. Q1			0.65 (0.41–1.00)							
**Colorectal cancer (*n* = 8)**																											
**Brown et al. **[12•] North America, Cancer and Leukemia Group B (CALGB) now Alliance for Clinical Trials in Oncology 89803		1024 patients with colon cancer stage III enrolled in a RCT of post-operative chemo-therapy, 56% male, median age: 60 y			4 m (midway through adjuvant treatment) and 14 m after surgery (6 m after treatment end)			7.3 y			Validated, semi-quantitative FFQ			394 DFS events, 350 RFS events, 311 OS events		Age, sex, race, performance status, T stage, positive lymph nodes, location of primary tumor, treatment arm, time-varying body mass index, physical activity, total energy, and mutual adjustment for whole grain and refined grain intake			Grain intake (servings/day)		DFS			RFS			OS
																			Refined grain (≥ 3 vs. < 1)		1.56 (1.09–2.24)			1.57 (1.08–2.30)			1.88 (1.25–2.85)
																			Whole grain (≥ 3 vs. < 1)		0.89 (0.66–1.20)			0.97 (0.71–1.33)			0.86 (0.62–1.20)
																			Subst.^c^		0.87 (0.79–0.96)			0.86 (0.77–0.96)			0.87 (0.78–0.97)
**Fadelu et al. **[32] North America, CALGB 89803 (Alliance)		826 patients with colon cancer stage III enrolled in a RCT of post-operative chemo-therapy, 62% male, median age: 66 y			14 m after surgery (6 m after treatment end)			6.5 y			Validated, semi-quantitative FFQ			238 DFS events, 199 RFS events, 177 OS events		Energy intake, age, sex, depth of invasion through bowel wall, number of positive lymph nodes, baseline performance status, treatment group, BMI, physical activity, aspirin use, glycemic load			Nut intake		DFS			RFS			OS
																			Total nut intake (≥ 2/week vs. never)		0.58 (0.37–0.92)			0.70 (0.42–1.16)			0.43 (0.25–0.74)
																			Tree nuts (≥ 1/week vs. never)		0.54 (0.34–0.85)			0.56 (0.33–0.94)			0.47 (0.27–0.82)
																			Peanuts (≥ 1/week vs. never)		0.81 (0.53–1.23)			0.97 (0.61–1.53)			0.60 (0.37–0.98)
**Jiang et al. **[33] Germany, Darmkrebs: Chancen der Verhütung durch Screening (DACHS)		2051 participants with invasive CRC, stages I–III, 59% colon cancer cases, 60% male, mean age: 68 y			79 d (93% after surgery)			5.2 y			Blood draw; Serum flavonoid metabolite levels quantified by UPLC/MS method			475 deaths, 254 due to CRC, 400 recs		Age, sex, stage, cancer site, BMI, education, physical activity, screening detected tumor, chemotherapy, diabetes, CVD, constipation, interval between surgery and blood draw, interval Between surgery and blood draw			Serum flavonoid conc		OM			CRC-M			Rec
																			Genistein Q4 vs. Q1 linear		1.00 (0.77–1.30) 1.03 (0.90–1.19)			0.83 (0.58–1.19) 0.96 (0.80–1.15)			0.98 (0.72–1.34) 1.05 (0.89–1.25)
																			Luteolin Q4 vs. Q1 linear		1.19 (0.92–1.53) 1.12 (0.89–1.40)			1.05 (0.74–1.47) 0.96 (0.70–1.32)			1.02 (0.76–1.36) 0.99 (0.75–1.30)
**Lochhead et al. **[34] USA, Nurses’ Health Study (NHS) and Health Professionals Follow-up Study (HPFS)		1550 participants with CRC, 69% women, 31% men, stage I-III, 64% with colon cancer, 20% with rectal cancer, 16% unknown location, mean age: 66 y			29.5 m			14.9 y			Validated, semi-quantitative FFQ (returned between 1 and 4 y after diagnosis)			641 deaths, 176 due to CRC		one-carbon nutrient intakes, pre-diagnostic folate intake, age and year, BMI, family history of CRC, aspirin use, multivitamin use, smoking status, alcohol consumption, physical activity, tumor location and differentiation, time from diagnosis to questionnaire return			Folate intake (Q5 vs. Q1)		OM					CRC-M	
																			Total folate		1.04 (0.60–1.82)					0.87 (0.65–1.16)	
																			Food folate		0.99 (0.63–1.54)					1.08 (0.84–1.38)	
**Ratjen et al. **[14••] Germany, biobank popgen		1404 long-term CRC survivors (stages I–IV), 56% male, 47% with colon cancer, 42% with rectal cancer, 6% with unknown location, 17% with metastases, median age at diagnosis: 62 y			6 y			7 y			Validated, semi-quantitative FFQ			204 deaths		Sex, age at diet assessment, BMI, physical activity, survival time from CRC diagnosis until diet assessment, tumor location, metastases, other cancer, type of therapy, smoking status, alcohol intake, total energy intake, time x age, time x BMI, time x metastases			3 plant-based indices (PDI)		AC-M						
																			PDI Q4 vs. Q1 per 10-point increase		0.46 (0.29–0.75) 0.72 (0.57–0.91)						
																			hPDI Q4 vs. Q1 per 10-point increase		0.76 (0.51–1.14) 0.82 (0.67–1.01)						
																			uPDI Q4 vs. Q1 per 10-point increase		1.29 (0.84–1.98) 1.19 (0.96–1.48)						
																			Healthy plant food groups (Q5 vs. Q1)		AC-M						
																			Whole grains		0.68 (0.44–1.06)						
																			Fruits		1.12 (0.70–1.81)						
																			Vegetables		0.78 (0.49–1.24)						
																			Nuts		0.48 (0.31–0.75)						
																			Legumes		0.88 (0.53–1.48)						
																			Vegetable oils		0.78 (0.50–1.22)						
																			Less healthy plant food groups (Q5 vs. Q1)		AC-M						
																			Fruit juices		0.74 (0.49–1.13)						
																			Refined grains		1.24 (0.78–1.97)						
																			Potatoes		0.99 (0.63–1.55)						
																			Sweets and desserts		0.64 (0.38–1.06)						
**Song et al. **[15•] USA, Nurses’ Health Study (NHS) and Health Professionals Follow-up Study (HPFS)		1575 participants with CRC, stages I–III, 61% women, 39% men, 72% with colon cancer, 22% with rectal cancer, 6% un-specified, median age: 69 y			2.2 y			8 y			Validated semi-quantitative FFQ, every 4 y, first FFQ collected at least 6 m but no more than 4 y after diagnosis			773 deaths, 174 due to CRC		Age at diagnosis, year of diagnosis, tumor grade, subsite, pre-diagnostic fibre intake, alcohol consumption, pack-years of smoking, BMI, physical activity, regular use of aspirin, glycemic load, consumption of total fat, folate, calcium and vitamin D * mutually adjusted ** + adjusted for fibre intake			Fibre intake (from different sources*), whole grain intake		CRC-M					AC-M	
																			Total fibre Q4 vs. Q1 Per 5 g/d		0.54 (0.35–0.85) 0.78 (0.65–0.93)					0.64 (0.51–0.80) 0.86 (0.79–0.93)	
																			Cereal fibre Q4 vs. Q1 Per 5 g/d		0.57 (0.38–0.86) 0.67 (0.50–0.90)					0.69 (0.57–0.84) 0.78 (0.68–0.90)	
																			Vegetable fibre Q4 vs. Q1 Per 5 g/d		0.65 (0.44–0.98) 0.82 (0.60–1.13)					0.74 (0.61–0.91) 0.83 (0.72–0.96)	
																			Fruit fibre Q4 vs. Q1 Per 5 g/d		0.95 (0.61–1.46) 0.91 (0.64–1.28)					0.93 (0.76–1.13) 0.92 (0.78–1.08)	
																			WG Q4 vs. Q1 Per 5 g/d		0.50 (0.32–0.77) 0.72 (0.59–0.88)					0.75 (0.61–0.92) 0.88 (0.80–0.97)	
																			WG** Q4 vs. Q1 Per 5 g/d		0.57 (0.35–0.92) 0.77 (0.62–0.96)					0.81 (0.65–1.01) 0.91 (0.83–1.01)	
**Van Blarigan et al. **[35] North America, CALGB 89803 (Alliance)		992 patients with colon cancer stage III enrolled in a RCT of post-operative chemo-therapy, 43% female, mean age 60 y			3 and 15 m (during and 6 m after chemo-therapy)			7 y			Validated, semi-quantitative FFQ			299 deaths		Age, sex, energy intake, T-stage, number of positive lymph nodes, baseline performance status, treatment arm, smoking, aspirin use, and other ACS components (BMI, physical activity), other dietary factors, alcohol consumption			ACS dietary guidelines		OS						
																			Fruit and vegetable intake (servings/d) ≥ 5 vs. < 5		0.60 (0.38–0.94)						
																			% WG from total grains (Q4 vs. Q1)		0.65 (0.45–0.94)						
**Van Blarigan et al. **[36] North America, CALGB 89803 (Alliance)		1011 patients with colon cancer stage III enrolled in a RCT of post-operative chemo-therapy			3 and 15 m (during and 6 m after chemo-therapy)			7 y			Validated, semi-quantitative FFQ			386 DFS events (305 deaths, 81 recs)		Age, sex, energy, T-stage, number of positive lymph nodes, baseline performance status, treatment arm, BMI, physical activity, smoking, aspirin use, intake of protein, alcohol, fats other than the fat of interest			Vegetable fat intake (g/d) Q4 vs. Q1		DFS 1.17 (0.84–1.62)						
**Urinary track cancer (*n* = 4); bladder cancer (*n* = 1), prostate cancer (*n* = 3)**																											
**Jochems et al. **[39] England, Bladder Cancer Prognosis Programme (BCPP)		728 patients (80% male) with NMIBC (stages pTa, pT1, pTis), tumor grade I-III, mean age at diagnosis: 69 y, *n* = 389 with post-diagnosis dietary intake data		1 y			3.7 y			Semi-quantitative FFQ			144 recs; 221 multiple recs			Age at diagnosis, sex, smoking status, tumor stage, grade, size, and multiplicity; *additionally adjusted for re-resection of a bladder tumor		Fruit and vegetable consumption			Time to first rec					Time to multiple rec*	
																		Total fruit intake (> 1.5 vs. < 1 port./d)			0.65 (0.44–1.00)					1.02 (0.82–1.20)	
																		Total vegetable intake (> 2.5 vs. < 1.5 port./d)			0.77 (0.50–1.18)					0.96 (0.74–1.09)	
																		Total fruit + vegetable intake (> 4 vs. < 2.5 port./d)			0.65 (0.42–1.01)					1.00 (0.85–1.18)	
**Van Blarigan et al. **[37] USA, Physicians’ Health Study (PHS)		926 men with non-metastatic PC (stage T1-T3); mean age at diagnosis: 69 y		5 y			10 y			Validated FFQ			333 total deaths, 56 due to PC			Age at diagnosis, energy, time since diagnosis, treatment, modified D’Amico risk category, BMI, smoking status, alcohol intake, protein intake, animal fat and trans-fat intake		Vegetable fat intake (en%)			AC-M					PC-M	
																		Q4 vs. Q1 Cont.^d^: CHO Subst.^e^: animal fat			0.65 (0.45–0.93) 0.67 (0.47–0.96) 0.56 (0.38–0.80)					0.93 (0.41–2.14) 0.81 (0.35–1.91)	
**Wang et al. **[16] USA, Health Professionals Follow-up Study (HPFS)		4,346 men with non-metastatic PC		n.a			7.8 y for lethal PC, 10.3 y for fatal PC			Validated, semiquantitative FFQ, at baseline and every 4 y thereafter			1285 deaths, 359 lethal^3^ PC cases, 264 fatal^4^ PC cases			Age at diagnosis, time period, time since diagnosis to FFQ, energy, BMI, physical activity, smoking status, Gleason score, stage, treatment, * + PSA screening history, family history of PC, Ethnicity, height, history of diabetes, intake of multivitamin and supplements, tomato sauce and coffee intake, MED score; ** + + family history of diabetes, myocardial infarction, cancer, history of high blood pressure and elevated cholesterol		Nut intake			Lethal PC^f^*			Fatal PC^g^*			AC-M**
																		Total nuts (≥ 5 times/ week vs. < once/month)			0.88 (0.57–1.35)			0.62 (0.36–1.07)			0.66 (0.52–0.83)
																		Other nuts (≥ 5 times/ week vs. < once/month)									0.70 (0.5–0.95)
																		Peanuts (≥ 5 times/week vs. < once/month)									0.79 (0.59–1.06)
**Wang et al. **[38] USA, Cancer Prevention Study II (CPS-II) Nutrition Cohort		5643 men with non-metastatic PC, mean age at diagnosis: 72 y		2.9 y			9.6 y			Modified Willet FFQ			363			Age and calendar year at diagnosis, race, tumor extent, nodal involvement, Gleason score, history of pre-diagnosis PSA-testing, education, treatment, history of CVD, physical activity, smoking status, total dairy intake		Lycopene and tomato intake (Q4 vs. Q1			PC-M (All PC)			PC-M (Lower risk PC)			PC-M (High-risk PC^h^)
																		Dietary lycopene (mg/d)			1.22 (0.91–1.64)			1.22 (0.82–1.83)			0.96 (0.56–1.65)
																		Tomato (serving/week)			0.98 (0.69–1.40)			0.71 (0.41–1.23)			1.15 (0.65–2.03)
																		Total tomato products (serving/d)			1.03 (0.76–1.41)			0.93 (0.59–1.46)			1.00 (0.60–1.66)

^a^All quartiles reported, because results suggest a non-linear (U-shaped) association

^b^Cont.: Per doubling of biomarker concentration (ng/ml)

^c^Subst.: Substitutional—1 daily serving of refined grain with whole grain

^d^Cont.: Continuous—10% more daily calories from vegetable fat and 10% less from carbohydrate (CHO)

^e^Subst.: Substitutional—10% more daily calories from vegetable fat and 10% less from animal fat (nutrient density model), en%: Energy %

^f^Lethal PC: cases that metastasised to distant organs at diagnosis or follow-up or that caused PC-death

^g^Fatal PC: cases that caused PC-death

^h^high-risk PC: T3–T4 or Gleason score 8–10, or nodal involvement; according to the National Comprehensive Cancer Network guidelines

*FU* mean/median follow-up, *t* time since cancer diagnosis, *N* number, *HR* (95% CI) hazard ratio (95% confidence Interval), *FFQ* food frequency questionnaire, *RCT* randomized controlled trial, *BC* breast cancer, *CRC* colorectal cancer, *NMIBC* non-muscle invasive bladder cancer, *PC* prostate cancer, *AC-M* all-cause mortality, *BC-M* breast cancer specific mortality, *OS* overall survival, *DFS* disease-free survival, *CVD* cardiovascular disease, *CVD-M* mortality due to cardiovascular disease, *OC-M* mortality due to other causes, *Rec* recurrence, *CRC-M* CRC-specific mortality, *RFS* recurrence-free survival, *PC-M* prostate cancer-specific mortality. *y* year, *d* day, *m* month, *port.* portion, *WG* whole grain, *PDI* overall plant-based diet index, *uPDI* unhealthy plant-based diet index, *hPDI* healthy plant-based diet index, *UPLC-MS/MS* ultra-performance liquid chromatography-tandem mass spectrometry, *UPLC/MS* ultra-performance liquid chromatography-tandem mass spectrometry, *conc.* concentration, *CH* carbohydrates

Of 23 eligible primary studies [11••, 12•, 13•, 14••, 15•, 16, 23, 24, 25•, 26, 27•, 28, 29, 30•, 31•, 32–39], two articles were found by manual literature search [26, 28]. The articles were mainly related to BC prognosis [11••, 13•, 23, 24, 25•, 26, 27•, 28, 29, 30•, 31•], followed by CRC prognosis [12•, 14••, 15•, 32–36] and PC prognosis [16, 37–39]. This was to be expected, as these common cancer sites have high 5-year survival rates, and diet is already known to be an important factor for the prevention of these cancers. General characteristics of eligible primary studies are summarized in .

Table 2. The majority of studies was conducted in North America/USA [11••, 12•, 13•, 15•, 16, 24, 26, 28, 29, 31•, 32, 34–38], three studies in Germany [14••, 27•, 33], one in Denmark [23], one in England [39] and two in China [25•, 30•]. All identified studies were prospective cohort studies using multivariable Cox proportional hazard regression models, with time since dietary assessment as the underlying time scale. Most studies (*n* = 20) investigated all-cause mortality/overall survival [11••, 12•, 13•, 14••, 15•, 16, 23, 24, 25•, 26, 27•, 28, 29, 30•, 31•, 32–35, 37], 14 studies examined cancer-specific mortality [11••, 13•, 15•, 16, 23, 24, 25•, 26, 27•, 28, 33, 34, 37, 38], and eight studies analysed cancer recurrence [11••, 16, 23, 25•, 26, 27•, 33, 39]. Combined endpoints, including recurrence-free and disease-free survival, were addressed in two [12•, 32] and seven studies [12•, 16, 30•, 32, 33, 35, 36], respectively. All included studies verified cancer diagnosis and validated outcomes of prognosis. Dietary information was mainly assessed at least 1 year after diagnosis [11••, 13•, 14••, 15•, 24, 26, 27•, 28, 29, 30•, 31•, 32, 34, 37–39], two studies [25•, 33] assessed early-postdiagnosis diet (2–3 months after diagnosis) and three assessed diet during active chemotherapy [12•, 35, 36]. Among the 23 eligible primary studies, four studies [14••, 27•, 30•, 37] explicitly focussed on long-term cancer survivors, with exposure assessment at least 5 years after cancer diagnosis. Dietary assessment was conducted via validated FFQs in all studies except three biomarker studies that used blood samples for exposure assessment, investigating circulating phytoestrogen metabolites [27•] and folate [29] in association with BC prognosis and serum genistein and luteolin concentrations [33] in relation to CRC prognosis. Repeated dietary assessment at multiple time points was conducted by 14 studies [11••, 12•, 13•, 15•, 16, 23, 24, 25•, 26, 32, 34–36, 38], with dietary intakes being studied as cumulative average values. Moreover, five studies [13•, 15•, 16, 23, 28] considered changes in dietary intake between pre- and postdiagnosis assessments. Adjustment for prediagnostic intake of the exposure of interest was reported in four studies [13•, 15•, 23, 34]. Dietary intakes assessed via FFQ were adjusted for total energy intake [11••, 12•, 13•, 14••, 15•, 16, 23, 24, 25•, 26, 28, 30•, 31•, 32, 34–39] and selected clinical prognostic, lifestyle and socio-demographic factors. All studies considered aspects of cancer stage, but some studies did not assess information on cancer treatment [11••, 15•, 23, 34] or relied on self-reports [13•, 14••, 24, 26, 28, 31•]. All studies considered age at diagnosis/at dietary assessment as a covariate and nine studies [13•, 16, 23, 24, 26, 27•, 33, 34, 37] adjusted for time between diagnosis and dietary assessment. Four studies [16, 30•, 33, 38] adjusted for further comorbidities.

## Association Between Plant-Based Diets and Overall Cancer Prognosis

One meta-analysis on intake of fruit (*n* = 3) and vegetables (*n* = 4) regarding all-cause mortality in survivors of different cancer sites was identified [7]. No clear associations were observed (Table 1).

## Association Between Plant-Based Diets and Breast Cancer Prognosis

For BC prognosis, we identified meta-analyses on fruit and vegetable, carotenoid and fibre intake regarding overall survival (Table 1). There was no indication for an association between fruit and vegetable intake with overall survival of BC (summary hazards ratio and 95% confidence interval (SHR (95% CI)) for high vs. low intake of fruit and vegetable combined: 0.95 (0.73, 1.24); I^2^ = 17%, *n* = 2)). Similar associations were observed for overall survival when investigating fruit and vegetable separately (SHR (95% CI) for high fruit intake: 1.04; (0.77, 1.42); I^2^ = 41%; *n* = 3; and for high vegetable intake: 1.08 (0.75, 1.55); I^2^ = 60%; *n* = 3). A meta-analysis on cruciferous vegetable intake and overall survival also did not show an association (SHR (95% CI): 1.03 (0.90, 1.17); I^2^ = 0%; *n* = 2) [17]. In line with this observation, another meta-analysis did not find an association between carotenoid intake (a proxy for fruit and vegetable intake) and overall- or BC-specific survival [18] (Table 1). However, a recent meta-analysis by Jayedi et al. [20••] identified an association between higher fibre intake and improved BC prognosis (SHR (95% CI): for all-cause mortality: 0.70 (0.55, 0.89); I^2^ = 0%; *n* = 3 and for BC-mortality: 0.72 (0.51, 1.01); I^2^ = 0%; *n* = 3] [20••]. Due to their oestrogen-like effects, soy/isoflavones are of particular interest for BC prognosis and showed a tendency (but imprecisely estimated) towards better overall survival in a high vs. low meta-analysis (SHR (95% CI): 0.80; (0.62,1.04); I^2^ = 24%; *n* = 2) [22••]. In that systematic review, single study findings indicated that higher postdiagnostic soy and isoflavone intake tended to be inversely associated with BC-specific mortality (hazard ratio and 95% confidence interval (HR (95% CI)): 0.83 (0.64, 1.07)) and a decreased relative risk of recurrence (HR (95% CI): 0.75 (0.61, 0.92)) among both US and Chinese women [40].

Regarding plant-based dietary patterns, one US prospective cohort study was identified examining the association between long-term adherence to an overall plant-based diet index (PDI), a healthful PDI (hPDI) and an unhealthful PDI (uPDI) regarding BC prognosis. While for the overall PDI no associations could be observed, the hPDI was associated with a 7% reduced relative risk of all-cause death (HR (95% CI): 0.93 (0.83,1.05)) and a 17% lower relative risk of death due to other causes than BC (HR (95% CI): 0.83 (0.71,0.98)). Conversely, the uPDI was associated with increased relative risks of all-cause death and death due to other causes than BC (HR (95% CI): 1.07 (0.96, 1.20) and 1.20 (1.02, 1.41), respectively). No associations were observed between plant-based eating patterns and BC-specific endpoints [11••].

In the Nurses’ Health Studies (NHS and NHS II), women with cumulative average of postdiagnostic high fruit and vegetable intake had a decreased relative risk of all-cause mortality (HR (95% CI): 0.82 (0.71, 0.94)) (Table 2). Results indicated that especially a higher vegetable intake was associated with lower all-cause mortality (HR (95% CI): 0.84 (0.72, 0.97)), while no association was observed for fruit intake. Subgroup analyses also pointed to lower all-cause mortality risk with higher intake of cruciferous vegetables (HR (95% CI): 0.87 (0.76, 0.99)) and vegetables high in β-carotene (HR (95% CI): 0.80 (0.70, 0.91)). Findings from the NHS study cohorts provided further evidence that BC survivors should be encouraged to maintain a high intake of fruit and vegetables after diagnosis, as a decrease in total fruit and vegetable intake, or vegetables alone, by one or more servings/day from pre- to postdiagnosis was associated with a 14% (HR (95% CI): 1.14 (1.01–1.27)) and a 16% (HR (95% CI): 1.16 (1.02–1.30)) higher relative risk of all-cause mortality [13•]. Other studies did not find an association between fruit and vegetable consumption or whole grain intake and BC prognosis [23, 28]. A recently published study from China showed that nut consumption was associated with an up to 50% reduced relative risk of BC recurrence, metastasis or mortality. Total nut intake ≥ 17 g/week, compared to non-consumption, was inversely associated with overall survival (OS) (HR (95% CI): 0.74 (0.52, 1.05)) and disease-free survival (DFS) (0.48 (0.31, 0.73)). Stratified analyses showed that the association was more evident among participants with a higher total energy intake for OS and among participants with early stage (I–II) BC for DFS. The associations did not vary according to nut type (i.e. peanuts, walnuts, other nuts) [30•].

No association with BC prognosis was observed for plant-based protein, whereas a modest survival benefit was reported for total protein intake [26]. Recently, it has been shown that high postdiagnostic intakes of total carbohydrates (CHO) were associated with higher relative risk of BC-specific and all-cause mortality [41]. However, a recent study suggested that the sources of CHO might have different effects on BC prognosis [24]. While higher intake of CHO from vegetables was associated with a decreased mortality risk (HR (95% CI): 0.86 (0.75, 0.97)), CHO from fruit juices, refined grains or potatoes were associated with increased relative risk (HR (95% CI) for CHO from fruit juice: 1.15 (1.01, 1.30); for CHO from refined grains: 1.16 (1.02, 1.32); and for CHO from potatoes: 1.13 (0.99, 1.28)). Similar associations were observed for BC-specific mortality (Table 2).

Addressing the controversially discussed association between soy/isoflavone intake and BC prognosis, a recently published study indicated that a higher postdiagnostic dietary intake of isoflavone was associated with reduced all-cause mortality following a linear dose-response trend. The strongest association was observed for ≥ 1.5 compared to < 0.3 mg/d (HR (95% CI): 0.65 (0.41, 1.00)). In subgroup analyses, the association was stronger for women with ER-PR-tumours and women who did not receive hormone therapy in the past [31]. A study from Hong Kong reported favourable associations with prognostic outcomes only for moderate intake of soy isoflavone, but not for the highest intake [25•]. This association was stronger in pre-menopausal women, women with triple-negative tumours and women who received initial hormone treatment [25•]. Since the mean soy isoflavone intake of this Chinese study cohort was 8.5 mg/d in the early postdiagnosis period, moderate intake here may be comparable to a high intake in the North American population (mean intake of 1.8 mg/d) [31].

Results of a biomarker study from Germany of long-term postmenopausal BC survivors did not point to a clear overall relationship between circulating phytoestrogen metabolites in the blood and prognosis. However, there were some surprising findings on individual phytoestrogen metabolites: Higher concentrations of luteolin were associated with an increased relative risk of BC-specific mortality. In addition, higher concentrations of genistein and resveratrol were associated with a higher relative risk of BC recurrence [27•]. Another biomarker study indicated that higher circulating folate concentrations were related to decreased relative risk of all-cause mortality (HR (95% CI): 0.41 (0.19, 0.90)) [29].

## Association Between Plant-Based Diets and Colorectal Cancer Prognosis

We identified a recently published systematic review and meta-analysis on postdiagnostic intake of whole grains and all-cause mortality in CRC survivors [19••], indicating an inverse association (SHR (95% CI): 0.83 (0.69, 0.99); I^2^ = 0%; *n* = 3).

In a German prospective cohort study of long-term CRC survivors, Ratjen et al. [14••] investigated adherence to plant-based dietary patterns in relation to all-cause mortality. The overall PDI was related to a decreased relative risk of all-cause mortality (HR (95% CI) per 10-point increase in PDI: 0.72 (0.57, 0.91)). Moreover, findings indicated inverse associations (but imprecisely estimated) for the hPDI (HR (95% CI): 0.82 (0.67, 1.01)) and positive associations for the uPDI (HR (95% CI): 1.19 (0.96, 1.48)) (Table 2).

The importance of a healthy plant-based diet for improving overall survival was further supported by findings of the CALGB 89803/Alliance trial. For colon cancer survivors, intake of ≥ 5 servings of fruit and vegetables per day was associated with improved overall survival. In the same study, it was shown that the quality of consumed grains might play a role in colon cancer prognosis [12•, 35].

A higher consumption of refined grains (≥ 3 vs. < 1 serving/day) was associated with an 88% higher relative risk of overall death (HR (95% CI): 1.88 (1.25, 2.85)). Similar findings were observed for colon cancer recurrence and disease-free survival (DFS). No clear associations were observed for intake of whole grain with colon cancer prognosis. However, replacing a daily serving of refined grains with whole grains was associated with a 13–14% lower relative risk of overall mortality, recurrence and DFS, respectively (HR (95% CI): 0.87 (0.78, 0.97), 0.86 (0.77, 0.96) and 0.87 (0.79, 0.96)) [12•].

Findings from the NHS and HPFS cohorts in CRC survivors [15•] indicated that higher intake of whole grains was associated with better survival (HR (95% CI) per 5 g/d increase for all-cause mortality: 0.88 (0.80, 0.97) and for CRC mortality 0.72 (0.59, 0.88)). In the same study [15•], each increase in total fibre intake of 5 g/d was associated with a 14% lower relative risk of overall death (HR (95% CI): 0.86 (0.79, 0.93)) and a 22% decreased relative risk of CRC-specific death (HR (95% CI): 0.78 (0.65, 0.93)). The association was particularly present for cereal fibre for all-cause mortality (HR (95% CI): 0.78 (0.68, 0.90)) and CRC-specific mortality (0.67 (0.50, 0.90)). In addition, a 5 g/d increase in total fibre intake from pre- to postdiagnosis was associated with decreased all-cause and CRC-specific mortality.

High consumption of nuts, especially tree nuts, was also associated with a reduced relative risk of overall death and cancer recurrence in 826 patients with stage III colon cancer. Compared to non-consumers, participants who consumed ≥ 2 servings of nuts per week had a HR (95% CI) of 0.43 (0.25, 0.74) for OS and of 0.58 (0.37, 0.92) for DFS. Subgroup analyses showed that the beneficial effects of nut intake were particularly attributable to tree nut intake [32]. However, postdiagnostic vegetable fat intake was not clearly associated with cancer recurrence or mortality in the CALGB 89803 trial (HR (95% CI) for DFS comparing high vs. low intake: 1.17 (0.84, 1.62)) [36].

The hypothesis that higher folate intake after CRC diagnosis might increase mortality risk after tumour resection was not supported in the NHS and the HPFS cohorts (HR (95% CI) for high vs. low total folate intake for overall mortality: 1.04 (0.60, 1.82) and for CRC-specific mortality: 0.87 (0.65, 1.16)) [34].

A biomarker study provided little evidence that postdiagnosis serum concentrations of flavonoid phytoestrogens, for which anticarcinogenic effects have been found in experimental studies, were associated with CRC prognosis [33]. Neither serum genistein (isoflavone) nor luteolin (flavone) were associated with overall mortality, CRC-specific mortality, CRC recurrence and/or disease-free survival (Table 2). However, the association might be different according to adjuvant chemotherapy received.

## Association Between Plant-Based Diets and Urinary Tract Cancer Prognosis

We identified one study on non-muscle invasive bladder cancer prognosis that did not clearly show a protective role for postdiagnosis fruit and vegetable consumption regarding recurrence [39]. In addition, for men diagnosed with non-metastatic PC, there is some, albeit very limited, evidence that a plant-based diet after diagnosis could improve overall survival. A higher intake of vegetable fat was associated with a decreased relative risk of overall death in the in the Physicians’ Health Study (HR (95% CI) for high vs. low intake: 0.65 (0.45, 0.93)). Additionally, replacing 10% of daily calories from animal fat with vegetable fat was associated with a relative risk reduction for all-cause mortality of 44% (HR (95% CI): 0.56 (0.38, 0.80)). Higher levels of saturated fat intake were also associated with increased risk of death from all causes. No clear association was detected for PC-specific mortality (HR (95% CI) high vs. low intake of vegetable fat 0.93 (0.41, 2.14)), probably attributable to the low number of events (56 PC-specific deaths) in the cohort [37]. In the HPFS cohort, higher postdiagnostic intake of nuts (≥ 5 servings per week vs. < 1 serving per month) was associated with a 34% lower relative risk of overall death (HR (95% CI): 0.66 (0.52, 0.83)). The estimates for PC-specific outcomes also pointed to inverse associations but were imprecisely estimated ((HR (95% CI) for lethal PC: 0.88 (0.57, 1.35) and for fatal PC: 0.62 (0.36, 1.07)) [16].

As dietary lycopene has been inversely associated with the risk of incident PC, postdiagnostic lycopene intake was evaluated in relation to PC-specific mortality in the Cancer Prevention Study II Nutrition Cohort [38]. No overall associations could be observed using a single dietary measurement. However, when lycopene intake was assessed at two different postdiagnosis time points, average intake was inversely associated with PC-specific mortality but only in men at advanced stages (HR (95% CI): 0.41 (0.17, 0.99)). This finding is probably due to chance, but it is also possible that the use of data from two consecutive FFQs allowed the detection of an association that would have been obscured by measurement error if only data from one questionnaire had been used [38].

## Future Research Directions

Interpretation of the evidence for dietary factors as determinants of cancer prognosis is challenging because of the broad scope of this area of research and heterogeneity between studies. There is large heterogeneity regarding study populations, exposures, their assessment (especially the timing of dietary assessment), as well as differences in the outcomes under investigation. In the future, studies are needed that are specifically designed to investigate dietary factors and cancer prognosis. These should allow examining associations between diet and cancer prognosis, including changes in dietary behaviour from pre- to postdiagnosis, in well-described populations of cancer survivors.

The findings of this systematic review are based on observational studies that are susceptible to confounding and reverse causation. The presence of symptoms, disease or treatment effects, comorbidities and overall health status might influence cancer survivors’ diet, and there is a plethora of other known and potentially unknown determinants of cancer prognosis [42]. Therefore, efforts should be made to clarify these factors as comprehensively as possible so that they can be considered in future studies. Until then, it is crucial that at least the most important predictors of cancer prognosis are considered covariates, including cancer stage, grade, received treatment and comorbidities [9]. Repeated assessments of diet at multiple time points should be conducted, as this may reduce within-person variation and better represents a cancer survivors’ long-term diet [13•]. Recommendations for dietary modification after a cancer diagnosis must be supported by valid and reliable evidence. Therefore, to analyse whether postdiagnostic diet has an independent benefit on cancer prognosis, future studies need to consider dietary intake before diagnosis or dietary changes from pre- to postdiagnosis.

Regarding the role of plant-based diets in cancer prognosis, future studies should focus on the investigation of plant-based dietary patterns and of specific plant-based dietary approaches that exclude meat products or animal products per se (e.g. vegetarian or vegan diets). To explore which components of a plant-based diet are particularly important for cancer prognosis, individual food groups, foods, nutrients and bioactive compounds (e.g. fruit, vegetables, whole grains, nuts, soy, fibre, isoflavones and carotenoids) need further investigation.

## Conclusions

Even though the evidence for postdiagnosis plant-based diets in association with cancer prognosis is very limited and studies are very heterogeneous, some preliminary conclusions can be drawn. Study findings suggest that adhering to plant-based diets might be beneficial for overall survival of cancer survivors (Table 1). The findings emphasise the importance of considering the quality of a plant-based diet for cancer survival, as healthy vs. unhealthy plant-based dietary patterns show associations in the opposite direction with mortality in BC and CRC survivors [11••, 14••]. There is initial evidence that healthy/unprocessed plant-based foods, including whole grains, nuts, fruit and vegetables may be beneficial for cancer prognosis. A high nut intake was consistently related to better survival of CRC, BC, and PC (Table 3), and, thus, further studies are needed to confirm these results. Preferring whole grains over refined grains and a high fibre intake after diagnosis seem to improve CRC prognosis. This is consistent with strong evidence that consuming whole grains and foods containing fibre decreases the risk of incident CRC [43]. The role of soy and isoflavone intake in BC prognosis remains unclear and needs further evaluation in large prospective studies from different geographic regions evaluating the whole spectrum of soy foods. To date, there is preliminary evidence that a moderate soy/isoflavone consumption has a favourable effect on prognosis, and there is no evidence that high soy consumption is associated with adverse prognostic outcomes in BC survivors. It is important that further studies on BC survival investigate whether the associations between dietary factors and survival vary according to hormone receptor status/tumour subtype (e.g. triple negative BC), hormone therapy and menopausal status, as this may contribute to the development of targeted dietary recommendations. Promoting vegetable fat intake, particularly in substitution of animal fat, might be beneficial for overall survival after a diagnosis of PC. The potentially beneficial role of dietary lycopene intake to improve PC survival needs further investigation.

**Table 3:** Summary findings of the associations between plant-based dietary factors and prognosis by cancer site

Exposure	Summary findings				
	Breast cancer	Colorectal cancer	Prostate cancer	Bladder cancer	Overall cancer
**PDIs**	Non-BC mortality ↓ for higher hPDI and ↑ for higher uPDI [11••]	AC-M ↓ for higher PDI, AC-M (↓) for higher hPDI and AC-M (↑) for higher uPDI [14••]	-	-	-
**Fruit and vegetables**	↔ from meta-analyses [17, 21]; AC-M ↓ for high intake of fruits and vegetables, and vegetables; AC-M and BC-M ↑ for high fruit juice intake; AC-M ↓ for high intake of cruciferous vegetables and vegetables high in β-carotene [13•]; ↔ for fruit and vegetable score [28]	OS ↓ for fruit and vegetables intake of 5 servings/d [35]	-	↔ [39]	↔ from meta-analysis [7]
**Whole grains (WG)**	↔ [23]; ↔ for % WG of total grains [28]	From meta-analysis [19••]: AC-M ↓ for high WG intake; AC-M and CRC-M ↓ for high whole grain intake [15•]; OS ↓ for preferring WG over refined grains [35]	-	-	-
**Refined grains**	-	DFS, RFS and OS ↑ for high refined grain intake; DFS, RFS, and OS ↓ for replacing 1 serving/d of refined grains with WG [12•]	-	-	-
**Nuts**	DFS ↓ for high nut intake [30•]	DFS and OS ↓ for high total nut intake, DFS, RFS and OS ↓ for high tree nut consumption [32]; AC-M ↓ for higher nut intake [14••]	AC-M ↓ for high total nut intake, and for other nuts than peanuts, Fatal PC (↓) [16]	-	-
**Carbohydrates (CHO)**	BC-M and AC-M ↑ for high CHO intake from fruit juice BC-M (↓) and AC-M ↓ for high CHO intake from vegetables, AC-M ↑ for high CHO intake from refined grains; BC-M ↑ and AC-M (↑) for high CHO intake from potatoes [24]	-	-	-	-
**Vegetable fat**	-	↔ [36]	AC-M ↓ for high vegetable fat intake and for replacing en% CHO or animal fat with vegetable fat [37]	-	-
**Vegetable protein**	↔ [26]	-	-	-	-
**Fibre**	From meta-analysis [20••]: AC-M ↓ and BC-M (↓) for high fibre intake	AC-M and CRC-M ↓ for high total fibre, especially cereal fibre, AC-M ↓ for vegetable fibre [15•]	-	-	-
**Soy/isoflavone**	From meta-analysis [22••]: OS (↓); AC-M, BC-M and Rec ↓ for moderate soy isoflavone intake [25•]; AC-M (↓) for high isoflavone intake [31]	-	-	-	-
**Phytoestrogens/flavonoids**	Rec ↑ for high conc. of circulating genistein and resveratrol; BC-M ↑ for high conc. of luteolin [27•]	↔ for serum genistein and luteolin conc [33]	-	-	-
**Folate**	OS ↓ for higher plasma total folate conc. [29]	↔ for high total and food folate intake [34]	-	-	-
**Carotenoids, lycopene**	↔ from meta-analysis [18]	-	-	↔ for lycopene but PC-M ↓ for cumulative high intake of lycopene among men diagnosed with high-risk PC [38]	-

↔ no associations with cancer prognostic outcomes observed, ↓ decreased risk, ↑ increased risk, (), only tendency (the one is included in the 95% CI), - no study available

*AC-M* all-cause mortality, *ACS* American Cancer Society, *BC-M* breast cancer mortality, *conc*. concentration, *CRC-M* colorectal cancer mortality, *DFS* disease-free survival, *OS* overall survival, *PC-M* prostate cancer mortality, *PDI* overall plant-based diet index, *uPDI* unhealthy plant-based diet index, *hPDI* healthy plant-based diet index, *Rec* recurrence, *RFS* recurrence-free survival, *WG* whole grain

In conclusion, this systematic review shows that plant-based diets and their components might have the potential to improve cancer prognosis, especially for breast, colorectal and prostate cancer survivors. Large well-designed cohort studies, considering important clinical factors (e.g. stage, treatment) and methodological aspects (e.g. time point of dietary assessment, dietary changes) are needed to provide more robust evidence on this topic.
